# Mepitel-Film zur Prävention der Radiodermatitis bei Brustbestrahlung

**DOI:** 10.1007/s00066-024-02217-7

**Published:** 2024-02-21

**Authors:** Cas Stefaan Dejonckheere, Ulrike Höller, Leonard Christopher Schmeel

**Affiliations:** 1 Klinik für Strahlentherapie und Radioonkologie, Universitätsklinikum Bonn, Venusberg-Campus 1, 53127 Bonn, Deutschland; 2 Deutsche Gesellschaft für Radioonkologie, 10117 Berlin, Deutschland

## Hintergrund

Nach initial widersprüchlichen Ergebnissen über die Wirksamkeit von Mepitel-Film zur Radiodermatitisprophylaxe im Rahmen der Brustbestrahlung in zwei randomisierten Phase-II-Studien [1, 2] führten Behroozian et al. eine randomisierte Phase-III-Studie durch, um die präventive Effektivität abschließend zu beurteilen [3].

## Methoden

In dieser multizentrischen kanadischen Studie wurden Patientinnen mit Risikofaktoren für die Entwicklung einer Radiodermatitis (RD) aufgenommen (Patientinnen mit großen Brüsten nach brusterhaltender Therapie, das heißt Brustumfang ≥ 91 cm oder Cup-Größe ≥ C, und Patientinnen nach Mastektomie) und im Verhältnis 2:1 randomisiert für Mepitel-Film im Bereich der gesamten Brust bzw. der gesamten Thoraxwand vs. Standardhautpflege mit einer Creme auf Wasserbasis (*„aqueous cream“*), jeweils ab Beginn der Strahlentherapie bis zwei Wochen nach deren Abschluss. Topische Kortikoide oder Antibiotika waren nach Maßgabe der Behandler in beiden Gruppen bei Beschwerden unter der Therapie erlaubt.

## Ergebnisse

In die *Intention-to-treat*-Analyse wurden 376 Patientinnen inkludiert (251 Mepitel-Film-Arm; 125 Kontrollarm). Der primäre Endpunkt, die Entwicklung einer Grad-2- oder Grad-3-Radiodermatitis während oder bis 3 Monate nach Strahlentherapie, zeigte sich signifikant besser in der Mepitel-Gruppe (16% vs. 46%; OR = 0,20; *p* < 0,0001). Auch eine Grad-3-Radiodermatitis und feuchte Desquamation waren seltener mit Mepitel-Film (3% vs. 14%, OR = 0,19; *p* < 0,0002 bzw. 8% vs. 19%, OR = 0,36; *p* = 0,002). Darüber hinaus erwiesen sich auch die *„patient-reported outcomes“* als vergleichsweise besser mit Mepitel-Film, nämlich weniger Schmerzen (*p* = 0,001), Brennen (*p* = 0,004), trockene Desquamation (*p* = 0,009), Erythem (*p* = 0,001), Pigmentierung (*p* < 0,0001) und Ödem (*p* = 0,03). Topische Antibiotika wurden seltener in der Mepitel-Gruppe verschrieben (23% vs. 42%; *p* < 0,0001), topische Kortikoide wurden in beiden Gruppen vergleichbar häufig rezeptiert. 8 Patientinnen (3%) der Mepitel-Gruppe brachen die Studie vorzeitig ab (2 Ausschlag, 1 Juckreiz, 5 sonstige Gründe).

## Schlussfolgerung der Autoren

Mepitel-Film führt zu einer signifikanten Reduktion von Radiodermatitis, insbesondere bei Patientinnen mit einem hohen Risiko.

## Kommentar

Die häufigste akute Nebenwirkung der Brustbestrahlung ist die Radiodermatitis (RD). Trotz laufender Forschungsanstrengungen sind bisher nur wenig wirksame prophylaktische und therapeutische Optionen verfügbar. Darüber hinaus kann aus methodischen Gründen nur über wenige Substanzen eine sichere Aussage zur Effektivität getroffen werden, wodurch die Behandlung der RD weiterhin stark variiert.

Um die Effektivität von Mepitel-Film, einer Polyurethanfolie auf Silikonbasis, beurteilen zu können, wurde die Studie von Behroozian et al. mit einer Kohorte mit erhöhtem Risiko für eine RD durchgeführt – denn insbesondere das Brustvolumen gilt als Risikofaktor für die Entwicklung einer RD [6]. Die meisten Patientinnen (93%) erhielten eine hypofraktionierte Strahlentherapie und der Anteil, der einen Boost oder Bolus erhielt, war in beiden Gruppen vergleichbar (29% vs. 30% bzw. 14% vs. 12%). Wertvoll sind die erhobenen *„patient-reported outcomes“*, da es bei der ärztlichen Bewertung der RD oftmals Diskrepanzen zu den von den Patientinnen berichteten Beschwerden gibt, insbesondere hinsichtlich des Einflusses auf die Lebensqualität [7]. Jedoch war aufgrund der sichtbar auf der Haut aufgeklebten Folienverbände eine Verblindung nachvollziehbarerweise nicht möglich, was einen Bewertungsbias damit aber wahrscheinlicher macht. Objektive RD-Bewertungsmethoden, obwohl in diesem Kontext bereits validiert [4–6], wurden nicht angewendet. Es wurde auch nicht stratifiziert nach dem Hauttyp (z. B. analog Fitzpatrick): Hellere Hauttypen (I und II) sind ein prognostisch günstiger Faktor für die Entwicklung einer milden RD [6] und waren häufiger in der Mepitel-Gruppe vertreten (33% vs. 22%; *p* = 0,0556). Dies könnte das Ergebnis im Sinne der Intervention verfälscht haben. Auch dedizierte Angaben zu den verwendeten Bestrahlungstechniken bleiben offen.

Die bisher publizierten intrapatientenrandomisierten Studien mit Mepitel-Film zur RD-Prävention im Rahmen von Brustbestrahlungen waren teilweise inkongruent [1, 2]. Herst et al. (*n* = 78) berichteten über eine 92%ige Reduktion der RD (Graduierung nach kombinierter Radiation-Induced Skin Reaction Assessment Scale [RISRAS]) und eine vollständige Prävention feuchter Desquamationen unter Mepitel-Film, was allerdings von Møller et al. (*n* = 79), die eine verblindete Beurteilung der Haut durch die Ärzte durchführten, nicht gleichermaßen bestätigt wurde. In der letztgenannten Studie erwiesen sich die *„patient-reported outcomes“* (Schmerzen, Juckreiz, Brennen, Ödem, Empfindlichkeit) zwar über alle Patientinnen hinweg, die *„clinician-reported outcomes“* jedoch nur bei der konventionellen Fraktionierung mit Mepitel-Film als vorteilhaft.

Zwei vergleichbare rezente Serien mit Hydrofilm (Polyurethanfilm; [4, 5]) bestätigen den präventiven „benefit“ barriereschichtbildender Folienverbände bei der adjuvanten Ganzbrustbestrahlung. Sowohl bei der konventionellen Fraktionierung (*n* = 56) als auch bei der moderaten Hypofraktionierung (*n* = 74) zeigte sich eine signifikante Reduktion der Prävalenz und des Schweregrads sowie eine verminderte subjektive Beschwerdeintensität der RD (Schmerzen, Brennen, Juckreiz, Einschränkungen). Neben *„clinician-“* und „*patient-reported outcomes“* wiesen auch objektive Messverfahren (Spektrophotometrie) eine signifikant reduzierte Erythem- und Pigmentierungsintensität nach.

Nebenwirkungen waren selten, mild und selbstlimitierend. Lediglich ekzematöse Effloreszenzen und Juckreiz wurden beschrieben. Die vorausgehenden Arbeiten zu Mepitel-Film bei der Brustbestrahlung deuteten bereits auf eine gute Verträglichkeit und Compliance hin: Bei Herst et al. brachen 4%, bei Møller et al. 15% die Anwendung des Mepitel-Films vorzeitig ab. In anderen Körperregionen ist die Datenlage für die Anwendung von Mepitel-Film zur RD-Prophylaxe bislang deutlich schwächer, so im Kopf-Hals-Bereich. Aufgrund einer nur geringen Tolerabilität des Mepitel-Films im Kopf-Hals-Bereich wurde die Studie vorzeitig abgebrochen (46% konnten den Filmverband nicht dauerhaft tolerieren; [8]).

Herausforderungen bei der Anwendung von Mepitel-Film waren laut den Autoren eine suboptimale Adhärenz in den Lymphabflussregionen (axillär, periklavikulär) oder bei Patientinnen mit großen Brüsten, wo ein Ersatz des Mepitel-Films öfter erforderlich war. Auch werden zusätzliche Kosten durch das Aufkleben und den Zeitaufwand der täglichen Positionskontrollen durch geschultes Personal beschrieben. Die durchschnittlichen Kosten an Mepitel-Film lagen bei den Brustbestrahlungen bei ca. 70 €, bei Thoraxwandbestrahlungen bei ca. 55 €.

### Fazit

Die Datenlage hinsichtlich der RD-Prävention in der adjuvanten Ganzbrustbestrahlung mit Film- und Folienverbänden konsolidiert sich mit der aktuellen Publikation von Behroozian et al. weiter. Schlussfolgernd lässt sich sagen, dass barriereschichtbildende Verbände im Kontext der adjuvanten Brustbestrahlung bei Hochrisikopatientinnen, nicht zuletzt aufgrund eines günstigen Nutzen-Risiko-Verhältnisses, durchaus empfohlen werden können.

*Cas Stefaan Dejonckheere, Bonn*

*Ulrike Höller, Berlin*

*Leonard Christopher Schmeel, Bonn*
